# Supplementary material for: Jasmone Is a Ligand-Selective Allosteric Antagonist of Aryl Hydrocarbon Receptor (AhR)
Source: Int J Mol Sci. 2023 Oct 27;24(21):15655. doi: 10.3390/ijms242115655 (PMC10648586; doi:10.3390/ijms242115655)
Supplement: Supplementary file 1 [file ijms-24-15655-s001.zip › ijms-2646880-supplementary.pdf]

Jasmone is a ligand-selective allosteric antagonist of aryl hydrocarbon receptor (AhR)

Radim Vrzal, Adéla Marcalíková, Kristýna Krasulová, Lenka Zemánková, Zdeněk Dvořák

Department of Cell Biology and Genetics, Faculty of Science, Palacky University, Slechtitelu 27, 783 71 Olomouc, Czech Republic

Corresponding author: Radim Vrzal

Department of Cell Biology and Genetics

Faculty of Science, Palacky University Olomouc

Slechtitelu 27

783 71 Olomouc

Czech Republic

Email: radim.vrzal@email.cz

Tel.: +420-58-5634904; Fax.: +420-58-5634905

Keywords: jasmone; AhR; CYP1A1; LS180; HepG2; ChIP;

Running title: Jasmone antagonizes AhR

Supplementary methods:

Luciferase activity inhibition assay (Figure S1A)

Cell line AZ-AhR was treated with 10 nM TCDD for 24 hrs. Then, after cellular lysis, the lysate was combined with increasing concentration of jasmone (0.1-100 μg/mL) and incubated for 30 minutes at room temperature. After that, luciferase activity was measured using Tecan Infinite M200 plate luminometer (Schoeller Instruments, Czech Republic).

Cell viability assays (Figure S1B, C)

Cell lines HepG2 or LS180 were treated with increasing concentrations of jasmone (1-100 μg/mL) and/or DMSO (0.1%; v/v) for 4 or 24 hrs.

a) Thereafter, the medium was replaced by PBS with ***MTT*** (MTT = 3-(4,5-dimethylthiazol-2-yl)-2,5-diphenyltetrazolium bromide) in final concentration of 0.3 mg/ml. The solution was discarded after 30-40 minutes of incubation and replaced by DMSO for dissolution of the formazan crystals. Absorbance was measured at 570 nm with Infinite M200 (TECAN, Austria).
b) Then, the crystal violet staining procedure was performed according to the manufacturer's recommendations with minor modifications. After incubation of the cells for 4 or 24 hrs, the cells were washed once with 60 μL of 1x washing solution and then 30 uL of crystal violet was applied for 20 min at RT. Consequently, cells were washed five times with 60 μL of 1x washing solution and finally 150 μL of 100% methanol was applied. After several minutes at RT, the optical density was measured with Tecan Infinite M200 plate luminometer (Schoeller Instruments, Czech Republic) at 570 nm.

The results are expressed as % of negative control, i.e., medium with the solvent.

AheRePa cells – generation and reporter gene assay (Figure S3)

Mouse Hepa1c1c7 cells were transfected with the reporter plasmid pGL-4.27-DRE and selection process were performed as described elsewhere (Novotna et al., 2011). Hygromycin resistant population was then tested for the activation of mouse Ahr with TCDD and FICZ. After selection and amplification of single colony, AheRePa cells were incubated with TCDD (1 pM – 50 nM) and FICZ (1 pM – 1000 nM) for 4 or 24 hrs in order to calculate EC50s and EC80s, which were used for 4 hrs treatment with jasmone.

Supplementary data:

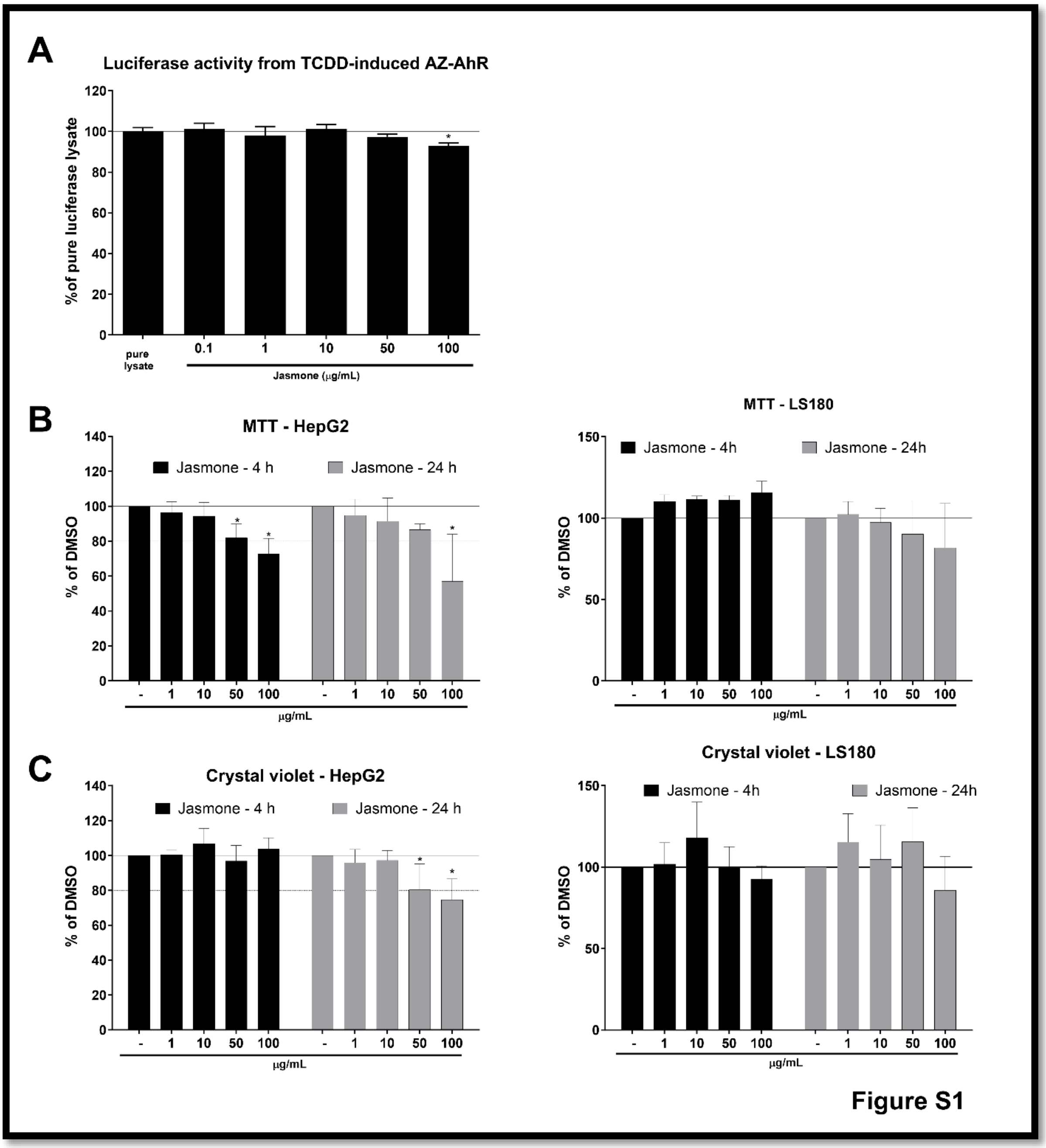

Figure S1:

Cell lysate from TCDD-induced AZ-AhR cells was incubated with jasmone for 5 minutes (**A**) and luciferase activity was determined. HepG2 or LS180 cells were incubated with jasmone (1-100 μg/mL) for 4 or 24 hrs and MTT (**B**) or Crystal assay (**C**) were performed. Data are expressed as % of pure TCDD-stimulated cellular lysate (**A**) or as % of negative control (**B**, **C**). They are the mean ± SD from measurements performed in quadruplicates (**A**) or the mean ±

SEM from at least 4 independent experiments (**B**, **C**). * - represents significantly different value ($p < 0.05$) from pure lysate (**A**) or negative control (**B**, **C**).

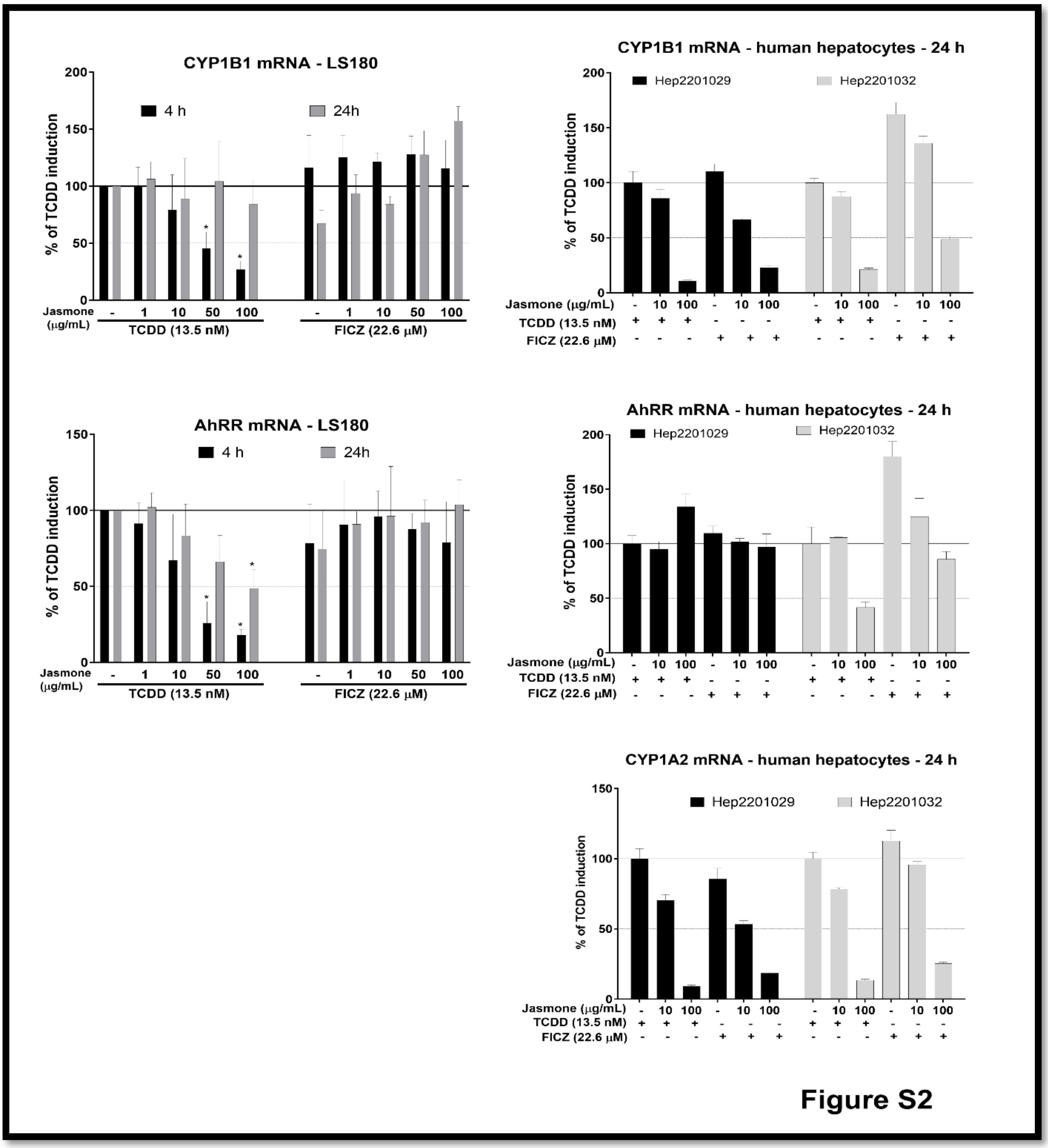

Figure S2:

LS180 cells or primary human hepatocyte cultures were treated with jasmone in the presence of TCDD or FICZ and CYP1B1, AhRR and CYP1A2 mRNAs were determined as described in *Materials and Methods* section. Bar graph express the % of TCDD induction. * - represents significantly different value ($p < 0.05$) from TCDD-treated sample.

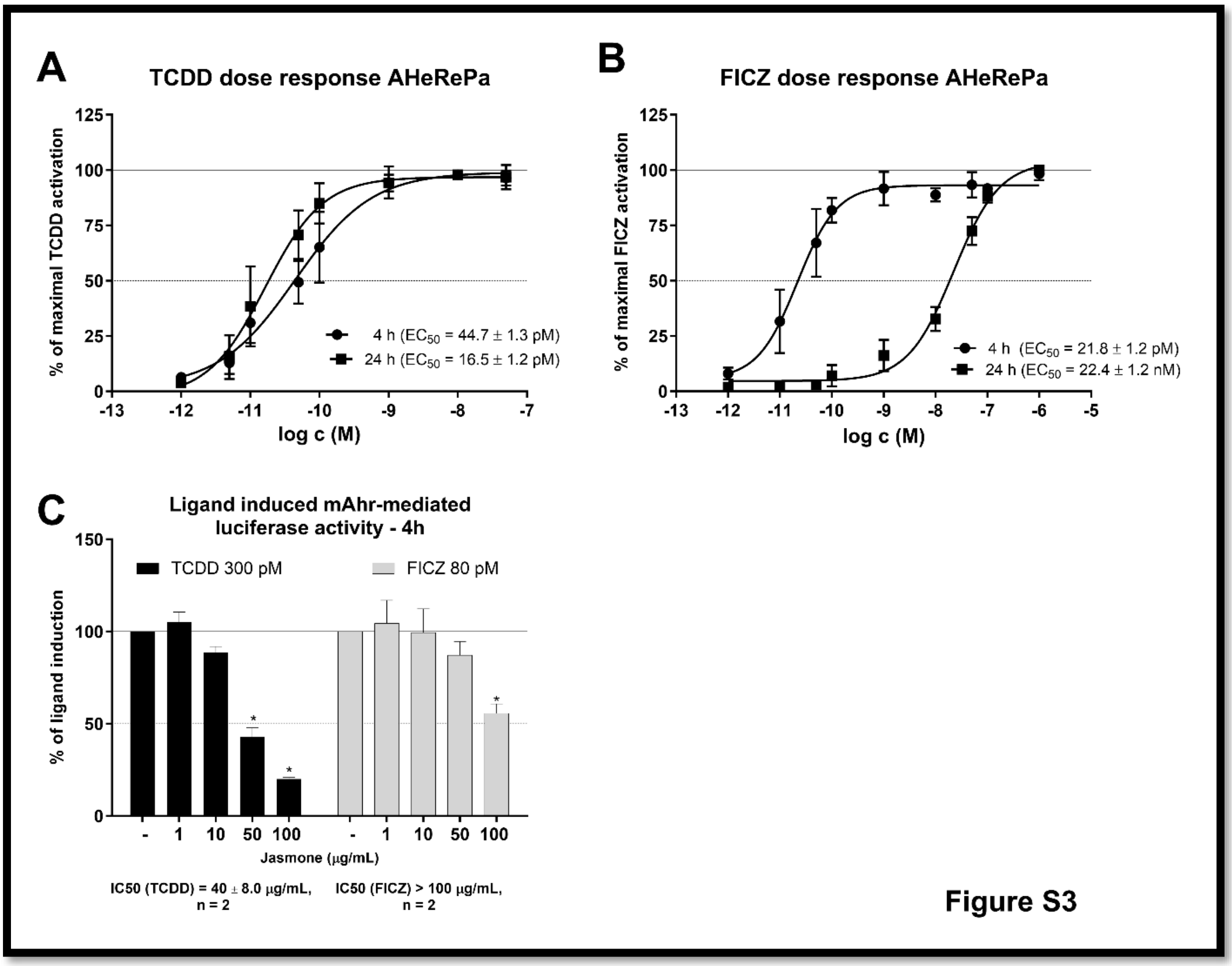

Figure S3

Dose-response analysis of murine Ahr-mediated luciferase activity in AheRePa cells after 4 and 24 hrs with (**A**) TCDD (0.001-50 nM) or (**B**) FICZ (0.001-1000nM). The effect of jasmone (1-100 µg/mL) on TCDD- and FICZ-inducible luciferase activity in murine AheRePa cells (**C**). * - represents significantly different value ($p < 0.05$) from TCDD- or FICZ-treated sample (**C**).

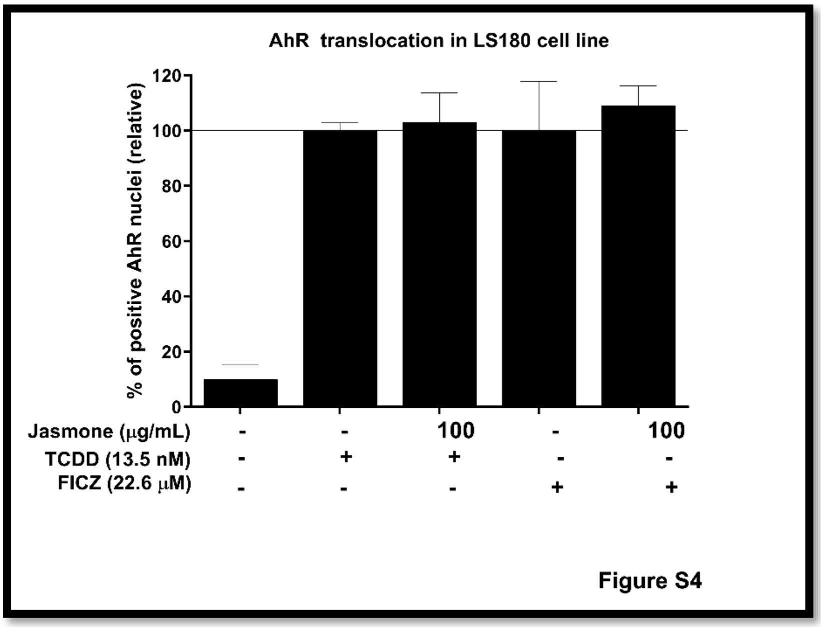

Figure S4

Quantitative analyses of AhR positive nuclei in LS180 cells incubated for 90 min with vehicle (EtOH 0.1% v/v) or jasmone (100 µg/mL) and TCDD (13.5 nM) or FICZ (22.6 µM).

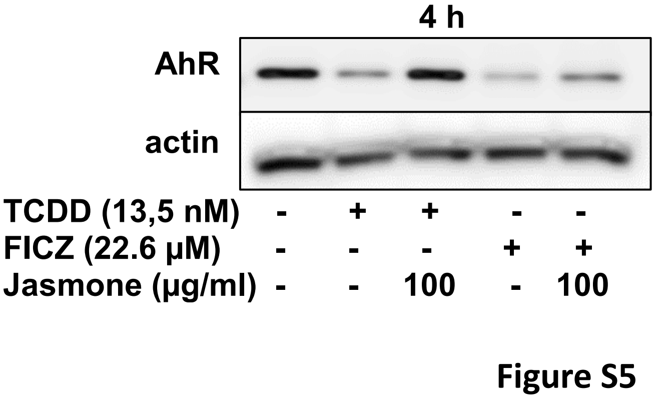

Figure S5

AhR protein level in LS180 cells co-treated with jasmone (100 µg/mL) in the presence of TCDD (13.5 nM) or FICZ (22.6 µM) for 4 hours.

References:

1. Novotna, A.; Pavek, P.; Dvorak, Z. Novel stably transfected gene reporter human hepatoma cell line for assessment of aryl hydrocarbon receptor transcriptional activity: Construction and characterization. *Environ. Sci. Technol.* **2011**, *45*, 10133–10139.
